# Advances and Potential New Treatments in Stroke Management

**DOI:** 10.1155/2014/120384

**Published:** 2014-02-17

**Authors:** Majaz Moonis, Padma Srivastava, Magdy Selim, Marc Fisher

**Affiliations:** ^1^UMass Memorial Medical Center, 55 Lake Avenue North, Worcester, MA 01655, USA; ^2^All India Institute of Medical Sciences, Ansari Nagar, New Delhi 110029, India; ^3^Beth Israel Deaconess Hospital, 330 Brookline Avenue, Boston, MA 02215, USA

The last 3 decades have brought more advances in stroke management than any other era before. This special issue is an attempt to highlight some of these areas ranging from stroke prevention to stroke rehabilitation. The validation of tPA in improving outcome in 1995 is perhaps one of the most important milestones achieved in acute stroke management [1]. However, there is a small but significant risk of symptomatic hemorrhagic conversion with such treatment. Dr. D. J. Blacker and his group describe some novel strategies to reduce the risk of hemorrhagic transformation after thrombolysis with minocycline as well as other strategies.

Recurrent stroke prevention is highly linked to identification of the underlying mechanism^2^. This still remains a problem in 25–30% of incident cases. Drs. M. Khan and D. J. Miller review the existing literature and discuss the current approaches to improve detection of mechanisms underlying these cryptogenic stroke cases in “*Detection of paroxysmal atrial fibrillation in stroke/TIA patients*.” Drs. V. Singh and N. J. Edwards, in their review article “*Advances in the critical care management of ischemic stroke*,” discuss the advances in acute stroke management including advances in critical care and endovascular treatment. Drs. P. B. Gorelick and U. Farooq in their review article “*Advances in our understanding of “resistance” to antiplatelet agents for prevention of ischemic stroke*,” provide an in-depth review of the current antiplatelet therapy and explore the concept of platelet resistance to antiplatelet agents, its place in clinical testing, and impact on outcomes.

Imaging techniques in acute stroke management continue to grow [2] and are addressed in context by Dr. P. Dubey and her colleagues in their review article “*Acute stroke imaging: recent updates*.” This includes the controversy of CT versus MRI based imaging and the current controversies and consensus.

Stroke recovery besides the natural history is impacted by several additional factors including comorbid conditions, the hospital course, and subsequent rehabilitation. In this context, Dr. B. Husaini and his group investigated the impact of comorbid depression in a large Medicaid group from Tennessee. They explored this often ignored, poorly understood factor and demonstrated its impact on length of hospital stay and outcome broken down by race and gender in “*Depression increases stroke hospitalization cost: an analysis of 17,010 stroke patients in 2008 by race and gender.*” The subsequent two articles on stroke rehabilitation explore the concept of recovery and neural plasticity (Drs. N. Takeuchi and S.-I. Izumi) and the intriguing role of piano playing in improving fine motor control (Drs. M. Villeneuve and A. Lamontagne). Fine motor control in the upper extremities is often the last modality to recover and a cause of persistent disability for many stroke patients.

We hope that highlighting these ongoing multifaceted efforts in our endless pursuits to improve stroke care will provide new insights to trigger more active research and to bring about new treatment strategies for ischemic stroke.

*Majaz Moonis* *Majaz Moonis* *Padma Srivastava* *Padma Srivastava* *Magdy Selim* *Magdy Selim* *Marc Fisher* *Marc Fisher*
